# Fatty acids intake in the Mexican population. Results of the National Nutrition Survey 2006

**DOI:** 10.1186/1743-7075-8-33

**Published:** 2011-06-08

**Authors:** Ivonne Ramírez-Silva, Salvador Villalpando, Jessica E Moreno-Saracho, Daniel Bernal-Medina

**Affiliations:** 1 Centro de Investigación en Nutrición y Salud. Instituto Nacional de Salud Pública, Cuernavaca Mor, México

## Abstract

**Background:**

There is growing evidence that quality, rather that quantity of fat is the determinant of cardiovascular risk. The objective of the study is to describe quantitatively the intake and adequacy of fatty acid classes among the Mexican population aged 5-90 years from a probabilistic survey.

**Methods:**

Dietary intake of individual and classes of fatty acids was computed from the dataset of the 2006 Mexican National Health and Nutrition Survey (ENSANUT2006), collected by a food frequency questionnaire. Adequacy was calculated in reference to authoritative recommendations.

**Results:**

The mean intake of total fatty acids (TFA ≈ 25%E) fell within WHO recommendations; the intakes of saturated fatty acids (SFA) among all age-groups (45-60%) and of trans fatty acids (TrFA) in 30% of school-age children and adolescents and 20% of adults exceeded international recommendations. The mean intake of polyunsaturated fatty acids (PUFA) and particularly of n6 and n3 PUFAS, was inadequately insufficient in 50% of the sample.

**Conclusions:**

The main public health concerns are the high intake of SFA and the suboptimal intake of PUFA in Mexican population. The TrFA intake represents a low public health risk.

## Background

The epidemiologic profile of México has changed in the past decades from a scenario dominated by infectious diseases to a new scenario of chronic non-transmissible diseases [1-7]. Cardiovascular diseases (CVD) and type 2 diabetes (T2D) are the principal causes of mortality in Mexico, followed by stroke [7,8]. In the last three Mexican National Health and Nutrition Surveys (1993, 2000 and 2006) hypoalphalipoproteinemia (prevalence = 60%) and hypertriglyceridemia (prevalence = 35%) were the most frequent cardiovascular risk (CVR) indicators in adult population [9-11]. This high prevalence of dyslipidemias in the Mexican population [12,13], results from several interacting factors, among which, the diet plays a key role. As by the Mexican National Health and Nutrition Survey of 2006 (ENSANUT-2006) the typical Mexican diet has a modest proportion of total fat (25% of the energy intake), and a high intake of carbohydrate (61%) [14]. Recent evidence postulates that CVR is associated with the quality rather than with the total intake of fat; saturated [15,16] and trans fatty acids are associated with the highest CVR [17,18]. A solid body of evidence has demonstrated that mono (MUFA) and polyunsaturated fatty acids (PUFA) reduce CVR [19,20]. The essential polyunsaturated fatty acids of the n-3 and n-6 families, have also important effects on the cardiovascular health [21]; mediated through the pro-(n-6) and anti-inflammatory (n-3) effects of their derived prostaglandins, tromboxanes and leucotrienes. It was postulated that the balance between these two families of compounds, rather than the absolute concentration, is determinant of their effect on the cardiovascular health [22]. However, recent evidence from two prospective cohorts demonstrated that the intake of, both, long-chain and intermediate-chain n-3 PUFA was associated with lower coronary heart disease (CHD) risk, not modified by n-6 PUFA intake [23] and that, both, alpha linolenic and linoleic acid intakes are associated with lower CHD risk [24].

There is little or null information about the intake of individual or classes of fatty acid in the Mexican diet, despite their risks and benefits for the health of the population.

The objective of the present analysis is to describe the distribution and adequacy of the dietary intake of individual and classes of fatty acids, representing protection or risk for cardiovascular diseases. This information is expected to give scientific support to the design of public health strategies and regulations helping to curve the epidemics of cardiovascular diseases in México.

## Methods

### Design and population

The information for the present analysis was obtained from the Mexican National Health and Nutrition Survey of 2006 (ENSANUT-2006) data set; this is a population-based, probabilistic survey, representative at the national level.

The probabilistic design of the survey was polyetapic, stratified and by conglomerates; a detail description was published elsewhere [25]. During the survey 48,304 households were visited, households to furnish dietary information were randomly selected from the list of the main sampling frame, aiming to accomplish 30% of the sample at each sampling site. The survey was powered to make inferences at national level by the following age groups: school-age (5-11 years), adolescents (12-19 years), adults (20-60 years) and over 60 years of age [25].

*Socio-demographic information*. Age, gender and socioeconomic level and household characteristics were extracted from the household questionnaire of the survey. For the purpose of this study, a principal components analysis (PCA) was performed on household characteristics (flooring material, ceiling, walls, water source, drainage, number of persons residing in the household and number of domestic appliances). The main factor extracted explained 40.4% of the total variance with a Kaiser-Mayer-Olkin (KMO) measure of sampling adequacy = 0.83 and was used as a proxy of socioeconomic status (SES). This factor had large loadings for household and community characteristics such as sewer system, indoor plumbing, refrigerator and television. Small loadings were observed for variables such as communal food distribution and number of people residing in the household. For the purpose of this article this factor was divided into tertiles and used as a proxy for low, medium and high socio-economic level [26].

### Dietary and food composition information

#### Dietary information

Dietary information was obtained using a food-frequency questionnaire (FFQ) collecting information on the 7 days prior to the survey. The FFQ included 101 food items for adults and adolescents and 100 for school-age children, organized in 12 "ad-hoc" selected food groups. The portion size and total number of servings, the number of days in a week, occasions in a day for each food item in the questionnaire consumed were registered in an electronic capture mask. The protocol and validation of the FFQ was published elsewhere [27].

#### Energy and macronutrients

The intake of energy and macronutrients was computed in two stages. In the first stage, the intake of individual foods in grams per day (g/day) was calculated, dividing the total amount by 7 days. In the second stage the intake in g/day was transformed into total energy, macronutrients and fatty acids intake using the food composition tables compiled by Instituto Nacional de Salud Publica, combining the USDA Food Composition Tables [28,29] and other tables from Mexico and Latin America [30,31].

#### Fatty acids intake

The intake of 38 individual fatty acids was estimated using the Fatty Acid Composition Tables for foods frequently consumed by the Mexican population, published by our group [32]. These tables include analytical information for more tan 300 foods, performed by gas chromatography. Total fat (TFA), is the result of weighing the fat extracted from food samples. The fatty acids were summed into their chemical classes, i.e. saturated (SFA) monounsaturated (MUFA), polyunsaturated (PUFA), omega 3 (n-3), omega 6 (n-6) and trans fatty acids (TrFA). We report here also the following individual fatty acids: myristic, palmitic, stearic, linoleic and alpha linolenic considered relevant for public health issues. Total fat, classes and individual fatty acids are reported as intakes in g/day, and as percentage contribution to the total energy intake (%E).

### Assessment of dietary adequacy

Adequacy of the intake of saturated and unsaturated fatty acids was assessed relative to the recommendations for the prevention of cardiovascular diseases published by the World Health Organization (WHO) [33]. The adequacy for n-3 and n-6 polyunsaturated fatty acids in school-age population was referred to the Dietary Reference Intakes (DRI) of the Institute of Medicine of USA [34].

The reference recommendations for the intake of fatty acids were based on the acceptable intervals for the intake of fats (AIIF), expressed as the percentage contribution to the total energy intake (%E).

The AIIF used for this analysis were, for the whole range of age (5-90 years): total fatty acids (TFA) 15-30%E; saturated fatty acids (SFA) < 10%E, polyunsaturated (PUFA) 6-10%E; trans fatty acids (TrFA) < 1%E; monounsaturated (MUFA) was the calculated difference, (TF-(SFA + PUFA + TrFA)) [33]. The AIIF for adult population (18-60 years of age) for n-6 and n-3 were 5-8%E and 1-2%E, respectively [33]. For children 5-8 years of age 10%E and 0.9%E and for 9-12 years of age 12%E and 1.2%E for n-6 and n-3, respectively [34].

Dietary adequacy for total and polyunsaturated fatty acids was graded and stratified into the following 3 categories: I) Adequate intake, defined when the intake/recommendation ratio was equal to 1; II) Insufficiently inadequate intake was defined when the intake/recommendation ratio was < 1; and III) Excessively inadequate intake was defined when the intake/recommendation ratio was > 1. Dietary adequacy for saturated and trans fatty acids (TrFA) was graded and stratified into the following two categories: I) Adequate intake, defined when the intake/recommendation ratio was equal to 1; II) Inadequate intake defined when the intake/recommendation ratio was > 1. Dietary adequacy for n-3 and n-6 fatty acids was defined by its compliance with the recommended n-6/n-3 ratios, ranging 5:1 to 10:1 [35]. Thus, the n-6/n-3 intake ratio was categorized as "balanced" when it was within the recommended interval or "not balanced" when otherwise.

The adequacy for the intake of MUFA was not evaluated because there is not an agreed cut off value, since the recommendation for MUFA is the difference between the sum of percentages recommended for SFA plus PUFA from the percentage contribution of total fat to the energy intake (%E).

For the purpose of these analysis all the dietary intake data were stratified by age into the following categories: school-age children (5-11 years), adolescents (12-18 years), adults (19-59 years) and adults older than 60 years (> 60 years).

### Data analysis

Subjects with not plausible dietary information (n = 1971 or 10% of the overall sample); school age children (n = 693); adolescents (n = 330), adults (n = 835) and adults older than 60 years (n = 113) and pregnant or lactating women were excluded from the analysis. Non-plausible registries were defined: a) When the adequacy for energy or macronutrients was 5 standard deviations (SD) above the mean for the appropriate reference; b) individuals whose energy adequacy was below 25% of the reference mean [27].

### Statistical analysis

The adjusted means for fatty acids, in grams, and for the percentage contribution to energy intake were estimated for all age groups, using multiple regression models adjusting for age, gender and socioeconomic level. The intakes of energy and fatty acids were not normally distributed, then, they were log transformed before the analysis. The proportions of the sample fulfilling the AIIF (% adequacy) were estimated using logistic or ordinal regression models. All statistics were adjusted by the effect of the design of the survey, using the module SVY for complex samples of STATA software, v 9.0 (Stata Inc, College Station PA).

## Results

### Characteristics of the sample

The present analysis included valid dietary information on 8,690 school-age children; 7,731 adolescents, 16,366 adults and 3,687 adults older than 60 years, representing 15.4, 16.4, 49.0 and 10.7 million subjects in the population, respectively. The female/male ratio was equal to 1:1 in school-age children and adolescents and in adults 1:0.6, such a difference was corrected using an appropriate weighing factor. Quintile distribution of school-age children and adults was homogeneous, while in adolescents there was a 24% excess in the lowest quintile and a 15.6% deficit in the highest (data not presented).

### Intake of fatty acids and its percentage of contribution to the energy intake

#### School-age children

The adjusted mean for energy intake was 1396 Kcal/d (95% C.I. 1373,1420). The adjusted mean of total fatty acids intake was 39.5 g/d or 26.7%E. The mean intakes were 15.6 g/d or 11.4%E for SFA; 8.2 g/d or 5.9%E for PUFA and 13.1 g/d or 9.4%E for MUFA.

The mean daily intakes of some individual fatty acids were: 8.4 g/d or 6.2%E for palmitic; 3.1 g/d or 2.4%E for stearic and 1.2 g/d or 1.0%E for myristic. The adjusted mean intakes for essential n-6 PUFAs were: 4.5 g/d or 3.3%E, (97.4% of total n-6 intake was linoleic acid) and 0.3 g/d or 0.02%E for n-3 PUFAs (87% of the n-3 intake was alpha linolenic acid) and 0.5 g/d or 0.04%E for TrFA [Table 1].

**Table 1:** Daily energy and fatty acids intakes and percentage contribution to energy of Mexican school-age children and adolescents

	Daily intake		Percentage contribution to energy (%E)	
	
	Mean^1^	**95% C. I**.	Mean^1^	SEM
**School age children**				

Energy (Kcal)	1396	1373-1420	-	-
Fatty acids (g/day) Total fatty acids	39.5	38.5-40.4	26.7	0.16
Saturated total	15.6	15.1-16.0	11.4	0.08
Myristic	1.2	1.2-1.3	1.0	0.01
Palmitic	8.4	8.2-8.6	6.2	0.04
Stearic	3.1	3.0-3.2	2.4	0.02
Polyunsaturated	8.2	8.1-8.4	5.9	0.04
n-6 PUFA	4.5	4.4-4.6	3.3	0.02
			**Contribution to n-6 PUFA**	
Linoleic	4.4	4.3-4.5	97.4	0.03
n-3 PUFA	0.3	0.28-0.3	0.02	0.00
			**Contribution to n-3 PUFA**	
Linolenic	0.3	0.2-0.3	86.8	0.20
Trans fatty acid	0.5	0.46-0.5	0.4	0.01
Monounsaturated	13.1	12.8-13.4	9.4	0.06
				

**Adolescents**				

Energy (Kcal)	1662	1632-1691	-	-
Fatty acids (g/day) Total fatty acids	46.4	45.3-47.5	26.6	0.17
Saturated total	17.2	16.8-17.7	10.7	0.08
Myristic	1.3	1.2-1.3	0.9	0.01
Palmitic	9.4	9.1-9.6	5.8	0.04
Stearic	3.6	3.5-3.7	2.3	0.02
Polyunsaturated	10. 7	10.4-10.9	6.5	0.05
n-6 PUFA	5.5	5.3-5.6	3.4	0.03
			**Contribution to n-6 PUFA**	
Linoleic	5.3	5.2-5.5	97.5	0.03
n-3 PUFA	0.3	0.34-0.36	0.02	0.00
			**Contribution to n-3 PUFA**	
Linolenic	0.31	0.30-0.32	87.4	0.18
Trans fatty acids	0.5	0.52-0.56	0.4	0.01
Monounsaturated	15.4	15.0-15.8	9.4	0.06

^1^Adjusted means estimated by linear regression models, adjusted by age, gender, socioeconomic level and survey design.

#### Adolescents

The adjusted mean of energy intake was 1632 Kcal/d (95% C.I. 1632, 1691). The adjusted mean intakes were: 46.4 g/d or 26.6%E for total fatty acids; 17.2/d or 10.7%E for SFA; 10.7 g/d or 10.7%E for PUFA and 15.4 g/d or 9.4%E for MUFA.

The mean daily intakes for some individual fatty acids were: 9.4 g/d or 5.8%E for palmitic; 3.6 mg/d or 2.3%E for stearic and 1.3 g/d or 0.9E% for myristic. The adjusted mean for essential n-6 PUFAs, n-3 PUFAs, and TrFA were comparable to those observed in school-age children [Table 1].

#### Adults

The adjusted mean for energy intakes were 1655 Kcal/d (95% C.I., 1630, 1680) and 1334 kcal/d (95% C.I., 1306, 1371) for adults and adults older than 60 years, respectively. The adjusted mean intakes were: 43.5 and 34.4 g/d or 25.1 and 24.6%E for total fatty acids; 15.7 and 13.1 g/d or 9.9 and 10.3%E for SFA; 10.2 and 7.6 g/d or 6.3 and 5.8%E for PUFA; and 14.4 and 11.1 g/d or 8.9 and 8.6%E for MUFA, for adults and adults older than 60 years, respectively.

The adjusted mean intakes were 4.9 and 3.7 g/d or 3.1 and 2.8%E for essential n-6 PUFAs, and 0.3 and 0.2 g/d or 0.02%E for n-3 PUFAs for adults and adults older than 60 years, respectively. In both age groups, the intake of linoleic acid represented 97% of total n-6 PUFAs and of alpha linolenic acid 85 and 87% of the n-3 PUFAs, respectively. TrFA intakes were of about 0.4 g/d or 0.04%E, comparable between the two age groups [Table 2].

The food sources yielding more than 50% to the daily intake were in progressive order, for SFA, milk and milk derivates, wheat bread and cookies, cheese, meat and egg, traditional Mexican corn dishes, fried beans and fried chips; for PUFA, fried beans, wheat bread and cookies, meat and egg, fast food, fried rice and avocado and for MUFAS egg and meat, milk and milk derivates, sweet wheat bread, avocado, traditional Mexican corn dishes and tortillas in all age groups. Except for the adult group, for whom milk and milk derivates ranked in 5^th ^place.

Sugared bread

**Table 2:** Daily energy and fatty acids intakes and percentage contribution to energy of Mexican adults and adults older than 60 years

	Daily intake		Percentage contribution to energy (%E)	
	Mean^1^	**95% C.I**.	Mean^1^	SEM
**Adults**				

Energy (Kcal)	1655	1630-1680	-	-
Fatty acids (g/day) Total fatty acids	43.5	42.6-44.4	25.1	0.14
Saturated total	15.7	15.3-16.0	9.9	0.06
Myristic	1.2	1.1-1.2	0.8	0.01
Palmitic	8.3	8.1-8.5	5.3	0.03
Stearic	3.3	3.2-3.4	2.1	0.01
Polyunsaturated	10.2	10.0-10.4	6.3	0.04
n-6 PUFAS	4.9	4.7-4.9	3.1	0.02
			**Contribution to n-6 PUFA**	
Linoleic	4.7	4.6-4.8	97.4	0.03
n-3 PUFAS	0.3	0.3-0.32	0.02	< 0.01
			**Contribution to n-3 PUFA**	
Linolenic	0.27	0.26-0.27	86.9	0.17
Trans fatty acids	0.5	0.47-0.51	0.4	< 0.01
Monounsaturated	14.4	14.1-14.7	8.9	0.05

**Adults older than 60 years**				

Energy (Kcal)	1338	1306-1371	-	-
Fatty acids (g/day)	34.4	33.4-35.5	24.6	0.22
Saturated total	13.1	12.6-13.5	10.3	0.10
Myristic	1.1	1.0-1.1	1.0	0.01
Palmitic	6.9	6.6-7.1	5.4	0.05
Stearic	2.7	2.6-2.8	2.2	0.02
Polyunsaturated	7.6	7.3-7.8	5.8	0.06
n-6 PUFAS	3.7	3.5-3.8	2.8	0.03
			**Contribution to n-6 PUFA**	
Linoleic	3.5	3.4-3.7	96.9	0.07
n-3 PUFA	0.2	0.21-0.23	0.02	0.00
			**Contribution to n-3 PUFA**	
Linolenic	0.19	0.18-0.20	84.8	0.35
Trans fatty acids	0.4	0.38-0.42	0.4	0.01
Monounsaturated	11.1	10.77-11.53	8.6	0.08

^1^Adjusted means estimated by linear regression models, adjusted by age, gender, socioeconomic level and survey design.

### Adequacy of the fatty acid intakes

#### School-age children

**Figure 1:**
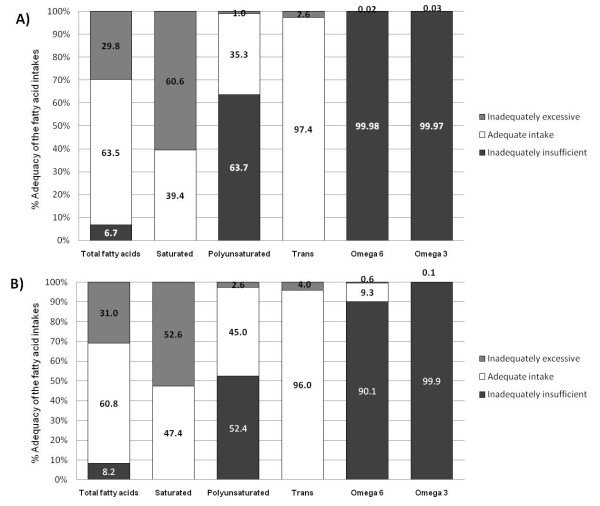
**Proportion of school-age children (A) and adolescents (B) as by their degree of adequacy in the intake of fatty acid classes**. Stratification for TFA and PUFA: I) Adequate intake, when the intake/recommendation ratio was equal to 1; II) Insufficiently inadequate intake when the intake/recommendation ratio was < 1; and III) Excessively inadequate intake when the intake/recommendation ratio was > 1. Dietary adequacy for saturated and trans fatty acids (TrFA) was stratified into the following two categories: I) Adequate intake, when the intake/recommendation ratio was equal to 1; II) Inadequate intake when the intake/recommendation ratio was > 1.

As by the AIIF, a proportion of 29.8% school age children had an inadequately excessive TFA intake (> 30%E); 63.5% had adequate (< 30%E), and 6.7% inadequately insufficient (< 15%E) intakes of TFA. About 60% of the school-age children had an excessively inadequate intake of SFA (> 10%E), 63.7% had an insufficiently inadequate intake of PUFA and 2.6% an excessive intake of TrFA. [Figure 1-A].

#### Adolescents

As by AIIF, a proportion of 31% adolescents had an inadequately excessive TFA intake (> 30%E); 51% had an adequate intake (< 30%E) and 18% an inadequately insufficient intake of TFA (< 15%E). About 50% of the adolescents had an excessively inadequate intake of SFA (> 10%E), 52% had an insufficiently inadequate intake of PUFA and 4.0% an excessive intake of TrFA. [Figure 1-B].

**Figure 2:**
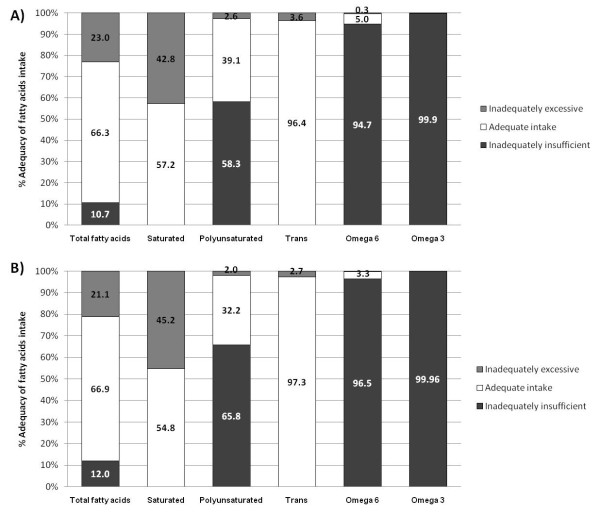
**Proportion of adults (A) and adults older than 60 years (B) as by their degree of adequacy in the intake of fatty acid classes**. Stratification for TFA and PUFA: I) Adequate intake, when the intake/recommendation ratio was equal to 1; II) Insufficiently inadequate intake when the intake/recommendation ratio was < 1; and III) Excessively inadequate intake when the intake/recommendation ratio was > 1. Dietary adequacy for saturated and trans fatty acids (TrFA) was stratified into the following two categories: I) Adequate intake, when the intake/recommendation ratio was equal to 1; II) Inadequate intake when the intake/recommendation ratio was > 1.

Adults and adults older than 60 years. As by the AIIF, a proportion of 31 and 23% of adults and adults older than 60 years, respectively, had an inadequately excessive intake (> 30%E); 66% of both groups had an adequate intake (< 30%E) and 12% an inadequately insufficient intake of TFA (< 15%E). About 42.8 and 45.2% of the adults and adults older than 60 years had an excessively inadequate intake of SFA (> 10%E), 59 and 64% had an insufficiently inadequate intake of PUFA, and 3.6 and 2.7% an excessive intake of TrFA. [Figure 2 A-B].

**Figure 3:**
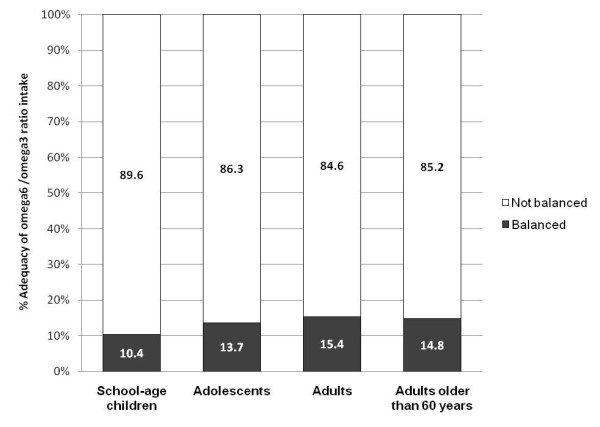
**Distribution of the Mexican population according to the fulfilment of recommendations of balanced intake of Omega6/Omega3**. Dietary adequacy for n-3 and n-6 fatty acids was defined by its compliance with the recommended n-6/n-3 ratios, ranging 5:1 to 10:1 [35].

The prevalence of abnormal n-6/n-3 ratios, was very high across all age categories, varying from 84.6% in adults to 89.6% in school-age children. The intake n-6 fatty acids exceeded by far that of n-3: (ratios: 15.7:1 for school-age, 15.9:1 for adolescents, 16.0:1 for adults and 16.7:1 for adults older than 60 years) contrasting with the range of recommended ratios 5:1-10:1. However the mean absolute intake of n-3 and n-6 classes was inadequately low in all age groups [Figure 3].

## Discussion

We present here evidence that across all age categories of the Mexican population the mean intake of total fat falls within the WHO recommendations for a healthy diet, but at least one third of the population have a total fat intake above the international recommendations. The third of the population with the TFA intake exceeding recommendations included mostly, adolescents living in urban areas (28%), in the 5th quintil of SES (24%) with no sex differences; and adults living in urban areas (24,7%), in the highest quintiles (4° and 5°) of SES (28 and 27%) and females younger than 40 years (64%) (data not shown).

We found an excessive intake of SFA combined with inadequately low intakes of PUFAS and of n-3 and n-6 fatty acids in particular, in about 60% of all age categories.

Compared with the total fat intake reported by the Mexican National Nutrition Survey of 1999 (30%) [36,37] there seems to be a reduction in the intake of total fat in 2006 (25%). However, most probably such a reduction was caused by methodological differences between the two surveys. The 1999 survey used the 24-h recall method, while the 2006 survey collected dietary information using a food-frequency questionnaire, known to underestimate the intake of energy and of fat in particular [38]. Most of the underestimation is believed to be related with uncertainties in estimating the amount of fat, specially cooking or baking fat hidden in prepared dishes. Thus, the absolute figures for total fat intake in the present report must be interpreted with caution, so we do not endorse any statement implying a reduction in the intake of fat in the Mexican population for the last 7 years.

The high intake of SFA was the most relevant indicator for cardiovascular risk in all age categories of the sample. At least half of the population ate more than 10%E as SFA. The foods contributing with 41% of the daily intake of SFA were, in a progressive order were bread and industrialized cookies, milk and milk derivates, egg and meat (data not presented).

The intake of PUFAs was below recommended for a healthy diet, about 60% of the population had an intake of PUFA below international recommendations, by so doing its protective role for CVR is seriously compromised. To comply with the WHO recommendation [33], an increment of 5%E as PUFAs would be desirable. Assuming that such an increment in the intake of PUFAs will displace isocalorically a similar amount of carbohydrates, to enhance the protective effect on cardiovascular risk of PUFA, as suggested in a recent meta-analysis [39]. The intake of n-6 and n-3 PUFAS was evidently below the recommended intakes by WHO (5-8%E and 1-2%E, respectively) in all age groups, recognizing that it represents a risk for the entire population. The intake of n-3 PUFAs (≈ 0.3%E) was the most inadequately low. A review reported that a high intake of n-6 PUFAs resulting in a high n-6/n-3 ratio is associated with higher risks for cardiovascular diseases, certain types of cancer and autoimmune diseases [22]. While a higher intake of n-3 PUFAs resulting in a lower n-6/n-3 ratio turned out to be protective. Authors reported that a 4:1 n-6/n3 ratio was associated with a 70% lower total mortality; ratios 2-3:1 reduced inflammation in patients with rheumatoid arthritis and a 5:1 ratio was beneficial for patients with asthma, while a ratio 10:1 increased adverse effects. Nevertheless, more recent evidence from two prospective cohorts, following very carefully the intake of n-6 and n-3 PUFAs, demonstrated in one study that, both, long-chain and intermediate-chain n-3 PUFA intakes were associated with lower CHD risk, without modification by the intake of n-6 PUFA [23], the other study found that, both, alpha linolenic and linoleic acid intakes are associated with lower CHD risk and that the combined intake of both fatty acids had synergistic effects [24].

It is probable that the combination of high SFA and low PUFA intakes might be associated with the high prevalence of hypercholesterolemia and hypertriglyceridemia reported for the same sample of Mexican adults from the ENSANUT-2006 [11]. Also this combination might be associated with the high cardiovascular morbid-mortality registered in Mexico during the last decade [40].

The information presented here is important from the programmatic point of view, because programs and strategies aiming to reduce the cardiovascular risk must be focused in reducing foods rich in saturated fatty acids and promoting the use of cooking oils with higher n-3 fatty acids content, beside increasing the consumption of fruit, vegetables and other healthy foods

The intake of TrFA contributed with 0.4%E, at the most, it was above the upper limit of intake in 4.0% of the population, suggesting that it represents a minimal public health risk. Nevertheless, actions taken by international and national health authorities, along with the food industry should not lessen their aims to eradicate TrFA from the national diet.

The main strength of the present study is its probabilistic design to be representative of the fatty acids intake in Mexican population. The mean intakes were adjusted for the design of the study, geographical distribution, socioeconomic level and other characteristics of the sample. The values were transformed logarithmically to achieve a normal distribution, procedure considered as the most appropriate by others [41]. Though, we found minimal differences in the results comparing transformed with not transformed data.

Another strength of this study is that the estimation of the fatty acid composition of food was based on a recent analysis by gas chromatography of the food items contributing to the 80% of the fat intake in Mexican population published by our group [32]. This accounts for cultural and regulatory differences in the content of fatty acids in modified and processed foods and cooking styles across countries [42].

Some weaknesses are to be considered also, the most relevant is that the dietary intake data were obtained from a food-frequency questionnaire and not by a 24-h recall method. However the FFQ was designed "ad hoc" and previously validated; the list of foods for the questionnaires were based on a 24-h survey applied to a probabilistic sample of Mexican population in 1999; such a list included food items consumed by 95% of the population and the serving sizes were standardized.

Due to the nature of the FFQ methodology, it has a greater degree of uncertainty compared with the 24-h recall method and cannot be ruled out some omissions of relevant foods in the list. Though, the memory bias is reduced in the FFQ, because there is a food list available and because encompasses a short period of time.

Further research is needed to explore the association among the intake of the fatty acids herein analyzed and biological markers of cardiovascular risk, as such the levels of lipids in plasma.

## Conclusions

In summary the dietary intake of saturated fatty acids is high and the intake of polyunsaturated fatty acids (PUFA) and in particular of n-3 and n-6 PUFA is low compared to WHO recommendations, representing a health risk for the Mexican population. Public policies should be enacted to reduce the intake of saturated fats by improving the quality of baking lard and promoting the consumption of defatted milk. These two foods are among the main sources of saturated fatty acids in the Mexican diet. Consumption of foods rich in n-3 and n-6 fatty acids (fish, nuts etc) are very low in the typical Mexican diet, thus alternatives like promoting a larger consumption of canola or soy bean oils or addition of n-3 fatty acids to cooking oils from other sources must be considered.

## Competing interests

The authors declare that they have no competing interests.

## Authors' contributions

IRS participated in the design and coordination of the study, performed the statistical analysis and interpretation of the data and drafted the manuscript. SV conceived the study, and participated in its design and coordination and helped to draft the manuscript. JEMS cleaned out the database information, carried out statistical analysis and revised critically the bibliography and the manuscript. DBM participated in the statistical analysis and revised critically the bibliography. All authors read and approved the final manuscript.
